# Point-of-Care Ultrasound-Directed Evaluation of Elbow Effusion

**DOI:** 10.5811/cpcem.2019.5.41674

**Published:** 2019-07-22

**Authors:** Peter Patitsas, Richard Davis, Robert Strony

**Affiliations:** Geisinger Medical Center, Department of Emergency Medicine, Danville, Pennsylvania

## Abstract

A 53-year-old male presented with pain in the right elbow that was sudden in onset and progressively worsening over approximately eight hours. The pain was exacerbated with any movement of the elbow. Of note, he had been recently admitted for robotic prostatectomy and had a prolonged hospital stay requiring a course of antibiotics. This case report details the emergency department evaluation of septic arthritis of the elbow with a focus on best practices for ultrasound- guided elbow arthrocentesis.

## INTRODUCTION

Septic arthritis results from an infection in a joint space from bacteria, fungus, virus, or even parasite. Delay in diagnosis of septic arthritis can have significant morbidity and even lead to death.^1^ While physical exam is critical to the diagnosis and evaluation of a septic joint, joint aspiration and culture is definitive.^2^ Because joint aspiration can be uncomfortable for a patient already presenting with joint pain, it is helpful to use ultrasound to evaluate whether there is an effusion. In the emergency department (ED), ultrasound is a critical diagnostic tool in the evaluation of patients in whom septic joint is clinically suspected. Ultrasound imaging can provide diagnostic information that expedites definitive diagnosis and treatment.

## CASE REPORT

A 53-year-old man presented to the ED with pain in his right elbow. The onset of this pain was sudden, was without a clear injury or event, and had been worsening for approximately eight hours prior to presentation to the ED. He denied trauma to the area recently or historically and had no prior surgeries to the right elbow. Of note, he did have a recent hospitalization for robotic-assisted prostatectomy due to prostate cancer and had been on a course of antibiotics during that hospitalization. The patient reported that the pain was exacerbated by any movement of the elbow, particularly with flexion to 90 degrees. There was mild associated swelling, but no significant redness or warmth. He denied fevers, chills, weakness, numbness, or paresthesia.

**Image 1:**
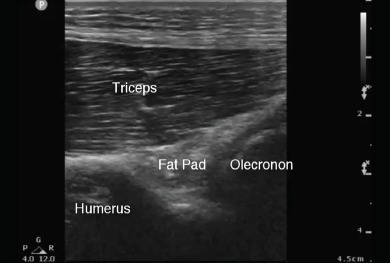
Posterior long-axis view of the elbow using a high-frequency linear transducer. The transducer indicator (p) is positioned superiorly and proximally. Note the normal elbow sonoanatomy and the position of the posterior fat pad to the elbow joint.

**Image 2:**
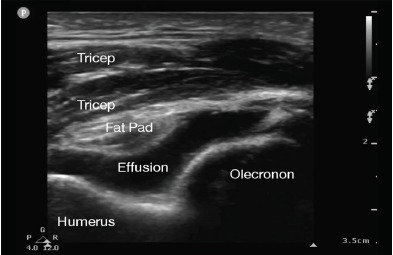
Posterior long-axis view of the elbow using a high-frequency linear transducer. The transducer indicator (p) is positioned superiorly and proximally. A large elbow joint effusion is labeled along with relevant anatomy. Note the posterior displacement of the fat pad.

**Image 3:**
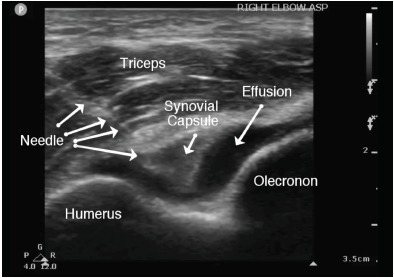
Posterior long-axis dynamic guided elbow aspiration using the high-frequency linear transducer and 18-gauge spinal needle. The transducer indicator (p) is again positioned superiorly and proximally. Note the posterior displacement of the synovial joint capsule into the effusion during aspiration.

The patient’s vital signs on presentation to the ED were within normal limits. He was well appearing and in no distress. On exam, we noted mild associated swelling of the right elbow, but no significant erythema or increased warmth. There was severe pain with any palpation of the right elbow. The right extremity was neurovascularly intact. Additionally, the patient was able to extend the affected wrist, abduct all fingers, and oppose the thumb. The differential diagnosis included hemarthrosis, elbow strain, septic arthritis, or crystalline disease. Complete blood count and basic metabolic panel were unremarkable. C-reactive protein was less than one milligram per liter and erythrocyte sedimentation rate was five millimeters per hour. Three radiographs of the right elbow were unremarkable without sail sign or posterior fat pad observed. We used a posterior oblique ultrasound approach of the right elbow to look specifically at the olecranon fossa. For the purposes of comparison, a normal sonographic appearance of the elbow (posterior approach) is depicted in Image 1. A joint effusion (Image 2) was identified between the olecranon fossa and its associated fat pad. We again used a posterior oblique approach to identify the joint effusion. Using an 18-gauge spinal needle with sterile technique, we aspirated the joint effusion under dynamic ultrasound guidance (Image 3). Six milliliters (ml) of cloudy, purulent fluid was aspirated.

Ultrasound helped to rapidly identify the effusion and guide the aspiration. Orthopedics was then consulted and expeditiously admitted the patient for suspected septic joint. He was treated with ceftriaxone and vancomycin. Studies of the joint aspirate showed white blood cell count (WBC) of 53,100 cells per cubic millimeter (mm^3^) and red cloudy fluid. There were no synovial crystals identified in the fluid.

In this case, ED point-of-care ultrasound (POCUS) quickly identified a right elbow joint effusion and was used for dynamically guided arthrocentesis of the right elbow joint.^3^ POCUS additionally allowed for exclusion of other etiologies for the patient’s symptoms such as tendon injury, muscle injury, or fracture, and these were not seen surrounding the elbow.^4^ Of note, while the initial analysis of the synovial fluid was suggestive of septic arthritis,^5^ given WBC >50,000 cells/mm^3^, cultures did not grow any bacteria. This was likely due to recent antibiotic use after prostatectomy, suggesting partially treated septic arthritis.

## DISCUSSION

This case is unique and relevant to the emergency physician (EP) because joint complaints are a common presentation that has a wide differential to consider. POCUS was essential because it allowed identification of an abnormal joint effusion and guided the elbow aspiration, which is difficult to do by landmark-only technique. Using POCUS, the EP can use three areas surrounding the elbow to evaluate a swollen elbow. These three areas are the olecranon fossa, coronoid fossa, and the radial fossa. Each of these has an associated fat pad. Normally, a fat pad fills these potential spaces, but if there is an effusion present (Image 2), the effusion displaces the fat pad. The posterior recess or olecranon fossa is the fastest and easiest area to explore. One can use either a long-axis or short-axis orientation of the ultrasound probe. It is also one of the easier approaches for aspirating a joint effusion.

**CPC-EM Capsule**

What do we already know about this clinical entity?

Septic joint is a well-characterized disease that usually occurs secondary to blood-borne infection.

What makes this presentation of disease reportable?

This patient had recently undergone prostatectomy and a course of antibiotics, with physical exam findings of elbow tenderness, mild swelling, and pain with movement of the joint.

What is the major learning point?

Ultrasound can be used to identify an effusion even when there is no erythema, warmth, or significant swelling over the elbow joint.

How might this improve emergency medicine practice?

Bedside ultrasound can supplement clinical suspicion for septic joint, aid in efficacy of joint aspiration, and expedite treatment at little associated risk and cost.

The elbow arthrocentesis (Image 3) should be performed with the patient sitting upright and at the appropriate height as to allow the sonographer comfort and to identify landmarks. A high-frequency linear transducer should be used and placed on the posterior aspect of the elbow with the indicator superior and proximally (toward the triceps body). Place the elbow in 90 degrees, arm abducted, with the patient’s forearm pronated using a side table to rest the hand. There are specific landmarks of the elbow that should be identified and palpated. Given that we used the posterior oblique approach and olecranon fossa for arthrocentesis, imagine a triangle formed by the lateral olecranon, the head of the radius, and the lateral epicondyle.

The skin should be prepared and cleaned with chlorhexidine. Next, using 1% lidocaine and a small needle (25 or 27 gauge), inject a wheal of local anesthesia into the dermis. With consistent pressure, identify the needle tip and advance the needle under sonographic dynamic guidance until the joint capsule is penetrated and joint fluid is aspirated. Collect synovial fluid, 5 ml to 10 ml, and remove the needle. Apply pressure with gauze for any bleeding observed and dress the puncture site. Send the specimen for testing.^6^

EPs can easily examine for the presence of elbow effusion with POCUS to aid in the diagnostic evaluation. The technique has little associated risk and cost. This bedside test can supplement clinical suspicion for septic joint, aid in efficacy of joint aspiration, and expedite treatment.

## CONCLUSION

A high index of suspicion for septic joint/arthritis is an important consideration for a patient presenting with joint pain significant enough to prevent decreased ability to move the affected joint. This case highlights that the classic symptoms of infection, including erythema, warmth, and swelling are not always present on physical exam especially when the patient has been on a recent course of antibiotics. POCUS is an excellent modality to identify and safely aspirate joint effusions. This patient proceeded to incision and drainage of the right elbow with orthopedics, and cultures continued to be negative. He made a full recovery. Using POCUS for workup and diagnosis of septic joint can improve efficacy of workup and time to treatment.
